# Circulating Serum MicroRNA-130a as a Novel Putative Marker of Extramedullary Myeloma

**DOI:** 10.1371/journal.pone.0137294

**Published:** 2015-09-21

**Authors:** Lenka Besse, Lenka Sedlarikova, Fedor Kryukov, Jana Nekvindova, Lenka Radova, Ondrej Slaby, Petr Kuglik, Martina Almasi, Miroslav Penka, Marta Krejci, Zdenek Adam, Ludek Pour, Sabina Sevcikova, Roman Hajek

**Affiliations:** 1 Babak Myeloma Group, Department of Pathological Physiology, Faculty of Medicine, Masaryk University, Brno, Czech Republic; 2 Department of Clinical Hematology, University Hospital Brno, Brno, Czech Republic; 3 Department of Hematooncology, Faculty of Medicine University of Ostrava and University Hospital Ostrava, Ostrava, Czech Republic; 4 Institute of Clinical Biochemistry and Diagnostics, Faculty of Medicine and Faculty Hospital in Hradec Kralove, Charles University, Hradec Kralove, Czech Republic; 5 Central European Institute of Technology, Masaryk University, Brno, Czech Republic; 6 Department of Internal Medicine—Hematooncology, University Hospital Brno, Brno, Czech Republic; Peking Union Medical College Hospital, CHINA

## Abstract

Poor outcome of extramedullary disease in multiple myeloma patients and lack of outcome predictors prompt continued search for new markers of the disease. In this report, we show circulating microRNA distinguishing multiple myeloma patients with extramedullary disease from myeloma patients without such manifestation and from healthy donors. MicroRNA-130a was identified by TaqMan Low Density Arrays and verified by quantitative PCR on 144 serum samples (59 multiple myeloma, 55 myeloma with extramedullary disease, 30 healthy donors) in test and validation cohorts as being down-regulated in myeloma patients with extramedullary disease. Circulating microRNA-130a distinguished myeloma patients with extramedullary disease from healthy donors with specificity of 90.0% and sensitivity of 77.1%, patients with extramedullary disease from newly diagnosed multiple myeloma patients with specificity of 77.1% and sensitivity of 34.3% in the test cohort and with specificity of 91.7% and sensitivity of 30.0% in the validation cohort of patients. Circulating microRNA-130a in patients with extramedullary myeloma was associated with bone marrow plasma cells infiltration. Further, microRNA-130a was decreased in bone marrow plasma cells obtained from patients with extramedullary myeloma in comparison to bone marrow plasma cells of myeloma patients without such manifestation, but it was increased in tumor site plasma cells of patients with extramedullary disease compared to bone marrow plasma cells of such patients (*p*<0.0001). Together, our data suggest connection between lower level of microRNA-130a and extramedullary disease and prompt further work to evaluate this miRNA as a marker of extramedullary disease in multiple myeloma.

## Introduction

Multiple myeloma (MM) is the second most common hematological malignancy characterized by clonal expansion of terminally differentiated plasma cells (PCs) producing monoclonal immunoglobulin (M-Ig). Accumulation of PCs leads to decrease of physiologic hematopoiesis in the bone marrow (BM) causing anemia and impairment of the immune system of MM patients and to further manifestation of the disease by osteolytic lesions, hypercalcemia and renal impairment [1,2].

Increasing knowledge of MM biology and improved management of the disease with novel agents, such as bortezomib, thalidomide and lenalidomide increased response rates and median overall survival (OS) of MM patients (pts). However, as MM treatment is usually not curative, most pts relapse. Such marked prolongation of OS has led to previously infrequent clinical presentation–relapses in extramedullary site [3,4]. Extramedullary disease (EM) is defined by presence of extraskeletal PCs infiltrates, either connected to the bone or infiltrating into soft tissues, such as liver, skin, lungs, central nervous system, genitourinary system, breast and pancreas [4–6]. EM can be found at the time of MM diagnosis (primary EM) or MM relapse (secondary EM) and generally is associated with aggressive progression, significantly shorter OS, poor prognosis for patients and treatment resistance [4,5,7–9].

MicroRNA (miRNA) are highly conserved, single-stranded, small (21–23 nucleotides long), non-coding RNA regulating gene expression and protein synthesis and playing a key role in fundamental biological processes, pathological events and tumorigenesis [10]. Moreover, miRNA were described as important diagnostic and prognostic markers in oncology, since their expression profiles were able to stratify pts and predict clinical outcome [11]. A novel class of miRNA, circulating miRNA, may be used as biomarkers in a minimally-invasive manner as they are easily obtained from various body fluids (serum, plasma, urine, etc.), stable and have been extensively studied in cancer [12–14]. As circulating miRNA profile has been associated with physiological or pathological conditions [15], it might provide valuable information in disease diagnosis, even EM.

MiRNA were repeatedly proven to play an important role in the pathogenesis of MM [16–18]; moreover, few studies showed circulating miRNA as putative biomarkers of MM [19–21]. Nevertheless, there is a complete lack of knowledge about circulating miRNA in EM. Thus, we aimed to define circulating serum miRNA deregulated in EM compared to MM and healthy donors in two independent cohorts of patients and their relation to clinically important parameters and cytogenetics.

To the best of our knowledge, this is the first study evaluating circulating miRNA with diagnostic potential for EM in such a large cohort of EM patients.

## Material and Methods

### Inclusion criteria and samples characteristics

In total, 144 peripheral blood (PB) serum samples were obtained from the Faculty Hospital Brno and Faculty Hospital Ostrava, Czech Republic, that included 55 serum samples from EM pts (5 primary EM, 50 secondary EM), 59 MM pts (49 newly diagnosed, 10 relapsed) and 30 healthy donors (HD). EM diagnosis was based on imaging and confirmed by biopsy and histopathological verification. PB serum samples were collected as follows: centrifugation 3500 rpm/15 min/20°C. Samples were frozen and stored at -80°C as 0.5 ml aliquots and thawed only once. For TLDA, 5 secondary EM, 5 newly diagnosed MM and 6 HD samples were used. As a testing cohort, 35 EM (3 primary EM, 32 secondary EM), 35 newly diagnosed MM and 30 HD samples were used; for validation, 20 EM samples (2 primary EM, 18 secondary EM) and specifically 23 newly diagnosed/relapsed MM samples (14 newly diagnosed, 10 in relapse) with PET/CT scan negative for EM were used. Also, miRNA analysis was performed on 13 samples of BMPCs: 7 relapsed MM pts and 6 secondary EM pts; and on 6 tumor samples of EM pts (all secondary EM; 5 bone related, 1 in soft-tissue related) with infiltration of PCs > 80%. BMPCs in the mononuclear cell fraction were enriched by anti-CD138+ immunomagnetic beads and sorted using AutoMACS (Miltenyi Biotec); samples with > 80% purity of PCs were used for miRNA/RNA extraction. Patients’ and donors’ characteristics are described (Table 1 and S1 Table). Samples were included only after patients signed the informed consent form approved by the Institutional Ethics Committee of University Hospital Brno and Institutional Ethics Committee of University Hospital Ostrava. All work was done according to the Declaration of Helsinki.

**Table 1:** Baseline characteristics of patients used in test and validation cohorts. Baseline characteristics of healthy donors (HD), newly diagnosed multiple myeloma (MM) patients and patients with extramedullary myeloma (EM) used in a test and validation phase of the study. P values show differences in clinical parameters between studied groups, significant differences are marked in bold.

	Test cohort			Validation cohort		P value
	HD	MM	EM	MM	EM	
No. of patients/donors	30	35	35	24	20	
Gender: males-females	50%-50%	43%-57%	63%-37%	38%-62%	50%-50%	0.274
Age median (min-max) [years]	55 (50–64)	70 (60–83)	58 (43–81)	64 (35–81)	64 (44–85)	**0.001**
ISS stage: I-II-III	ND	38%-24%-38%	56%-22%-22%	52%-38%-10%	11%-56%-33%	**0.004**
Durie-Salmon stage: I-II-III	ND	6%-20%-74%	4%-4%-92%	32%-27%-41%	6%-11%-83%	**0.001**
Durie-Salmon substage: A-B	ND	86%-14%	93%-7%	96%-4%	61%-39%	**0.006**
Ig isotype: IgG-IgA-IgM-IgD-LConly-NonSecr.-Biclonal	ND	51%-23%-6%-3%-9%-6%-3%	40%-28%-3%-9%-14%-6%-0%	63%-21%-4%-0%-8%-0%-4%	75%-15%-0%-0%-10%-0%-0%	0.473
Light chains: kappa-lambda	ND	70%-30%	51.5%- 48.5%	67%-33%	70%-30%	0.410
**Type of EM**						0.333
Bone related	ND	0%	48%	0%	57%	
Bone unrelated	ND	0%	36%	0%	14%	
Both types	ND	0%	16%	0%	29%	
**No. of previous treatment lines**						**0.001**
None	ND	100%	9%	58%	10%	
1-2-3-4-more	ND	0%	31%-29%-23%-8%-0%	25%-17%-0%-0%-0%	20%-25%-25%-10%-10%	
**Biochemical parameters: median–min-max)**						
Hemoglobin (g/l)	ND	102.0 (64.5–140)	109.5 (75.0–141)	119.0 (87.0–153)	107.5 (75.0–142)	0.054
Thrombocytes (count x109)	ND	220.0 (86.9–561)	193.5 (59.0–315)	179.0 (57.0–337)	177.5 (68.8–388)	**0.022**
Calcium (mmol/l)	ND	2.36 (1.85–3.59)	2.33 (1.78–2.84)	2.29 (2.10–2.86)	2.34 (2.05–3.13)	0.954
Albumin (g/l)	ND	39.7 (24.6–48.5)	37.5 (28.7–47.0)	39.8 (26.5–51.4)	36.2(19.9–43,8)	0.104
Creatinine (umol/l)	ND	91.0 (49.0–484)	92.5 (48.0–266)	76.0 (48.0–290)	101.0 (57.0–519)	0.141
β2-microglobulin (mg/l)	ND	3.72 (1.57–19.60)	3.23 (1.18–12.70)	2.61 (1.48–9.17)	4.87 (1.63–17.92)	**0.003**
Lactate dehydrogenase (ukat/l)	ND	3.09 (1.57–11.50)	4.93 (3.11–35.23)	3.60 (1.80–12.30)	3.74 (1.70–16.66)	**0.001**
C-reactive protein (mg/l)	ND	4.30 (0.0–161.50)	8.30 (1.50–97.70)	1.60 (0.90–17.00)	4.25 (0.6–87.3)	**0.003**
Monoclonal Ig (g/l)	ND	25.0 (0.0–64.7)	15.5 (0.0–51.1)	27.7 (0–50.0)	23.3 (0.0–60.0)	0.073
Plasma cell infiltration of bone marrow (%)	ND	18.0 (10.0–68.4)	4.4 (0–96.6)	12.8 (1.6–84.0)	29.0 (1.20–90.0)	0.023
**Chromosomal abnormality in BMPCs**						
13q14 deletion	ND	6 (23.1%)	16 (64.0%)	NA	NA	NA
17p13 deletion	ND	2 (7.7%)	7 (26.9%)	NA	NA	NA
1q21 gain	ND	6 (25.0%)	17 (68.0%)	NA	NA	NA
IgH disruption	ND	8 (57.1%)	15 (71.4%)	NA	NA	NA
Translocation t(4;14)	ND	1 (5.9%)	6 (30.0%)	NA	NA	NA
1p36 deletion	ND	1 (4.2%)	6 (30.0%)	NA	NA	NA
Hyperdiploidy	ND	11 (47.8%)	12 (46.2%)	NA	NA	NA

ND = not defined, NA = not available

### Extraction of RNA enriched for microRNA

Total RNA enriched for miRNA was extracted from all serum samples using miRNeasy Kit (Qiagen, Germany) modified for circulating miRNA according to manufacturer’s instructions. Tumor tissue was lysed using MagNa Lyser Green Beads on MagNa Lyser (Roche, Switzerland), then total miRNA/RNA was extracted from lysed tissue or BMPCs using miRVana miRNA Isolation Kit (Ambion, Life Technologies, CA, USA), according to manufacturer´s recommendation. MiRNA/RNA quantity was assessed by NanoDrop ND-1000 Spectrophotometer (Thermo Fischer Scientific, MA, USA) as described previously [20].

### TaqMan Low Density Arrays

Megaplex profiling using human TaqMan Low Density miRNA Arrays (TLDA) A+B, v.3.0 (Life Technologies, CA, USA) was performed to evaluate the expression of 667 miRNA as described previously [20]. Briefly, 60 ng of miRNA/RNA was reverse-transcribed into cDNA using TaqMan MicroRNA Reverse Transcription Kit and Megaplex RT Primers, v.3.0. Pre-amplification was performed using TaqMan PreAmp MasterMix and TaqMan PreAmp Primers, v.3.0 and pre-amplified product was loaded into the TLDA. Raw data were analyzed using SDS software version 2.3, RQ Manager v.1.2.1 (Life Technologies, CA, USA) as described previously [20].

### Candidate miRNA confirmation by qPCR

Individual TaqMan miRNA assays for 4 miRNA (hsa-miR-222-002276, hsa-miR-130a-000454, hsa-miR-34a-000426, hsa-miR-195-000494, Life Technologies, CA, USA) were used for qPCR on 7500 Real-Time PCR System. Reverse transcription and qPCR was performed as described previously [20]. Level of each serum miRNA was normalized based on the level of circulating miR-19b (hsa-miR-19b-000396; Life Technologies, CA, USA) using 2^-ΔCT^ equation, quantification of cellular miR-130a was based on the level of cellular RNU48 (cat no: 001006), using 2^-ΔCT^ equation. Repeatability of miRNA extraction and qPCR was also assessed (S1 Fig).

### Interphase fluorescence in situ hybridization analysis (I-FISH)

Routine I-FISH analysis of BMPCs in a test cohort of pts was available for 74.3% (26/35) of MM, and 56.4% (31/55) of EM samples, as described previously [22]. The following aberrations were studied: gain 1q21, deletion 13q14, deletion 17p13, disruption of IgH and translocation t(4;14). Hyperdiploidy status was determined by commercial probes mapping to chromosome 5 (LSI D5S23/D5S721), 9 (CEP9) and 15 (CEP15) (Abbott Molecular, Des Plaines, IL, USA).

### Statistical methods

TLDA data were analyzed according to manufacturer's recommendations. Relative expression levels of target miRNA were determined by the equation 2^-ΔCT^, calculated as: _Δ_C_T_ = C_T miR-of-interest -_ C_T miR-19b_. MiR-19b was chosen by GeNorm as a reference for normalization of miRNA expression levels as it was the most stable across all samples (M value according to GeNorm = 0.245). Relative miRNA levels were calculated with the RQ Manager 1.2. 2^-ΔCT^ normalized expression data from TLDA screening phase of the study were statistically evaluated in the environment of statistical language R by use of Bioconductor package and LIMMA approach combined with hierarchical clustering (HCL) [23,24]. For multiple testing, Benjamini-Hochberg correction was used to assess adjusted p value.

Standard descriptive statistics were applied in the analysis; median supplemented by min-max or interquartile range for continuous variables and absolute and relative frequencies for categorical variables. Statistical significance of differences in continuous variables among groups of pts was analyzed using nonparametric Kruskal-Wallis or Mann-Whitney U test. For the robust analysis of continuous parameters relationship, the Spearman correlation coefficient was adopted. The OS was determined according to International Myeloma Working Group guidelines. Cox proportional hazards models were used to assess the association of prognostic factors with overall survival. P-values below 0.05 were considered as statistically significant in all analyses. Data were statistically analyzed with IBM SPSS Statistics, v.20, R v. 2.15.3 and Exiqon GenEx software.

## Results

### Low Density Arrays study

**Fig 1:**
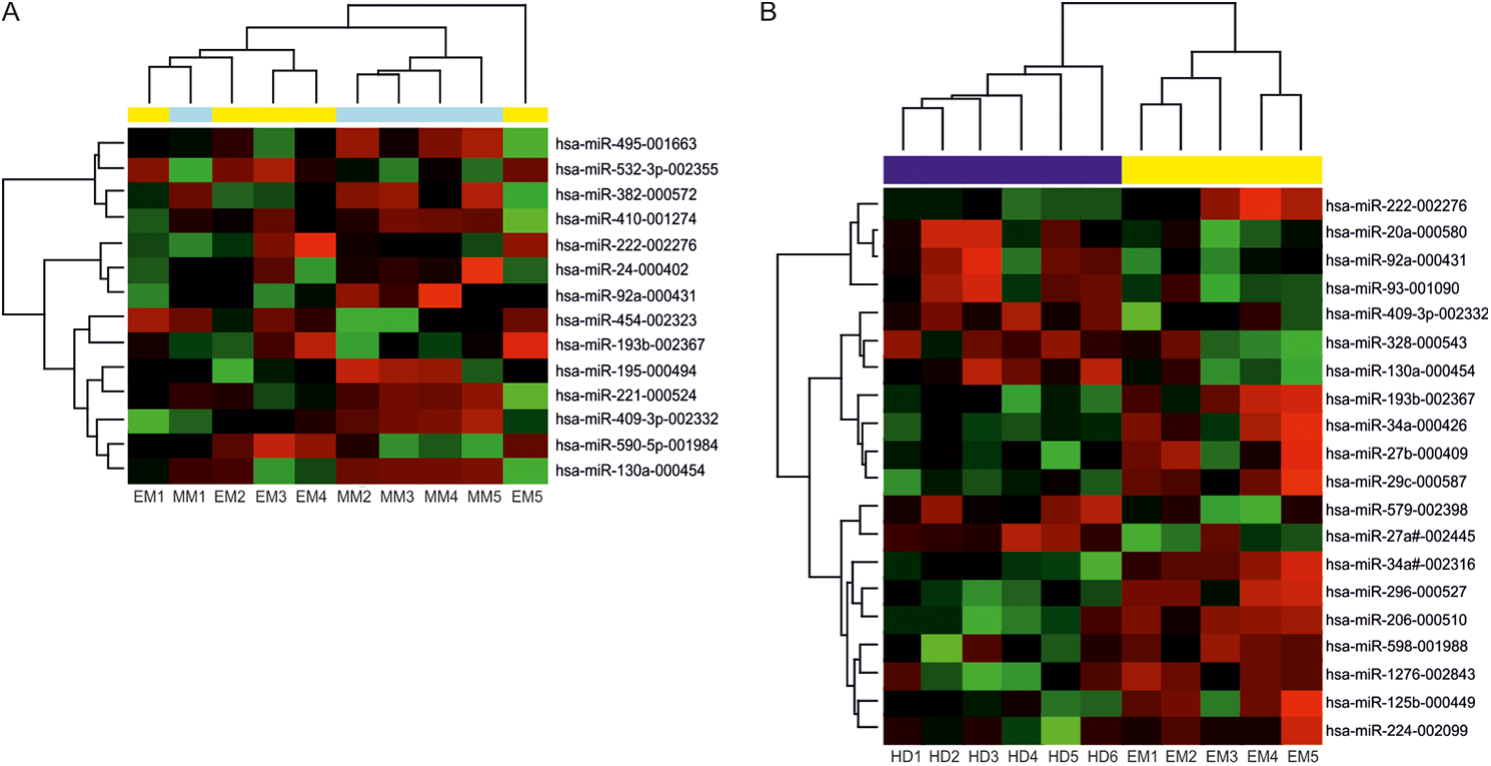
MicroRNA expression pattern in extramedullary myeloma (EM), multiple myeloma (MM) patients and healthy donors (HD). Hierarchical clustergram discriminating EM serum samples from A) MM, yellow color indicates EM serum samples, light blue MM serum samples, *p*<0.05. B) HD, yellow color indicates EM serum samples, dark blue indicates HD serum samples, *p*<0.05. Differential expression of miRNA is shown by the intensity of red (up-regulation) *versus* green (down-regulation).

Screening of 667 miRNA using TaqMan Low Density Arrays (TLDA) was performed on serum samples of 5 newly diagnosed MM pts, 5 secondary EM pts and 6 HD (S1 Table) to identify differentially expressed circulating miRNA. Only miRNA with Ct < 32 were taken into consideration. Fourteen miRNA were deregulated (all *p*<0.05, adjusted *p*<0.41) between MM and EM pts: 5 miRNA were up-regulated (miR-532-3p, miR-193b, miR-590-5p, miR-454 and miR-222) and 9 miRNA were down-regulated (miR-221, miR-382, miR-495, miR-130a, miR-409-3p, miR-24, miR-410, miR-195, miR-92a) in EM samples (Fig 1A). Further, 20 miRNA were on the top of the list of deregulated miRNA between EM and HD serum samples (all *p*<0.05, adjusted *p*<0.40), out of them 12 miRNAs were up-regulated (miR-34a*, miR-206, miR-193b, miR-222, miR-296, miR-34a, miR-29c, miR-1276, miR-125b, miR-598, miR-224 and miR-27b) and 8 miRNAs were down-regulated (miR-27a*, miR-130a, miR-579, miR-93, miR-20a, miR-92a, miR-93 and miR-409-3p) in EM samples (Fig 1B).

Although after correction for multiple testing, none of miRNA reached statistical significance, miR-222, miR-130a, miR-34a and miR-195 were chosen for verification by qPCR as they were the most differently expressed between the groups and showed the highest fold change and most favorable expression.

### Confirmation of candidate miRNA using qPCR in a test and validation cohort of patients

Since qPCR is more sensitive and quantitative over a wider dynamic range than TLDA, we employed miRNA specific assays (miR-222, miR-130a, miR-34a and miR-195) on a larger cohort of 35 EM pts, 35 newly diagnosed MM pts and 30 HD to test candidate miRNA expression between HD/MM/EM samples.

Although by qPCR, there was a trend for different expression for miR-195 and miR-222 between HD, MM and EM samples, observed also by TLDA, it did not reach statistical significance (*p* = 0.107 and *p* = 0.143, resp.), and therefore these miRNA were excluded from further studies. On contrary, miR-34a and miR-130a were differently expressed between the studied cohorts of samples (both *p*<0.0001), miR-34a was increased in EM samples and miR-130a was decreased. These data confirmed results of the screening phase (Table 2).

**Table 2:** MiRNA levels in a test cohort of patients. Data are presented as a median of normalized miRNA expression and interquartile range. Kruskal-Wallis test was used to compare the values. Fold change (FC) between extramedullary myeloma (EM) patients *versus* newly diagnosed multiple myeloma patients (EM/MM) and extramedullary myeloma patients *versus* healthy donors (EM/HD), and p values are presented. Significant values *p*<0.05 are marked with bold and italics.

miRNA	HD median (25–75)	MM median (25–75)	EM median (25–75)	EM/MM FC	EM/HD FC	P
**miR-222 **	0.192 (0.141–0.259)	0.227 (0.138–0.312)	0.243 (0.145–0.602)	1.071	1.266	0.143
**miR-130a **	0.100 (0.083–0.147)	0.057 (0.043–0.097)	0.053 (0.024–0.070)	0.920	0.530	***<0*.*0001***
**miR-195 **	0.328 (0.252–0.382)	0.324 (0.248–0.386)	0.271 (0.227–0.337)	0.836	0.826	0.107
**miR-34a **	0.004 (0.003–0.008)	0.009 (0.007–0.014)	0.018 (0.007–0.027)	2.000	4.500	***<0*.*0001***

To further prove that these miRNA can distinguish EM patients from MM patients with no EM, a validation cohort consisting of 20 primary and secondary EM pts and 24 MM pts (14 newly diagnosed, 10 in relapse) with active disease, but without EM verified by PET/CT was obtained. MiR-130a was significantly decreased in EM in comparison with MM samples (*p* = 0.012). Importantly, its levels were comparable with levels of EM pts from test cohort (*p* = 0.172). Also, miR-34a was decreased in EM in comparison with MM pts (*p* = 0.039), which is, however, opposite to the results obtained from previous cohort and therefore it cannot distinguish EM from MM pts (Table 3).

**Table 3:** MiRNA levels in a validation cohort of patients. Data are presented as median of normalized miRNA expression and interquartile range. Mann-Whitney U test was used to compare the values. Fold change (FC) between extramedullary myeloma disease (EM) patients *versus* multiple myeloma (MM) patients and p values are presented. Significant values *p*<0.05 are marked with bold and italics.

miRNA	MM median (25–75)	EM median (25–75)	EM/MM FC	P
**miR-130a**	0.122 (0.078–0.200)	0.064 (0.034–0.120)	0.525	***0*.*012***
**miR-34a **	0.035 (0.027–0.066)	0.020 (0.013–0.040)	0.571	***0*.*039***

### ROC analysis of miR-130a

**Fig 2:**
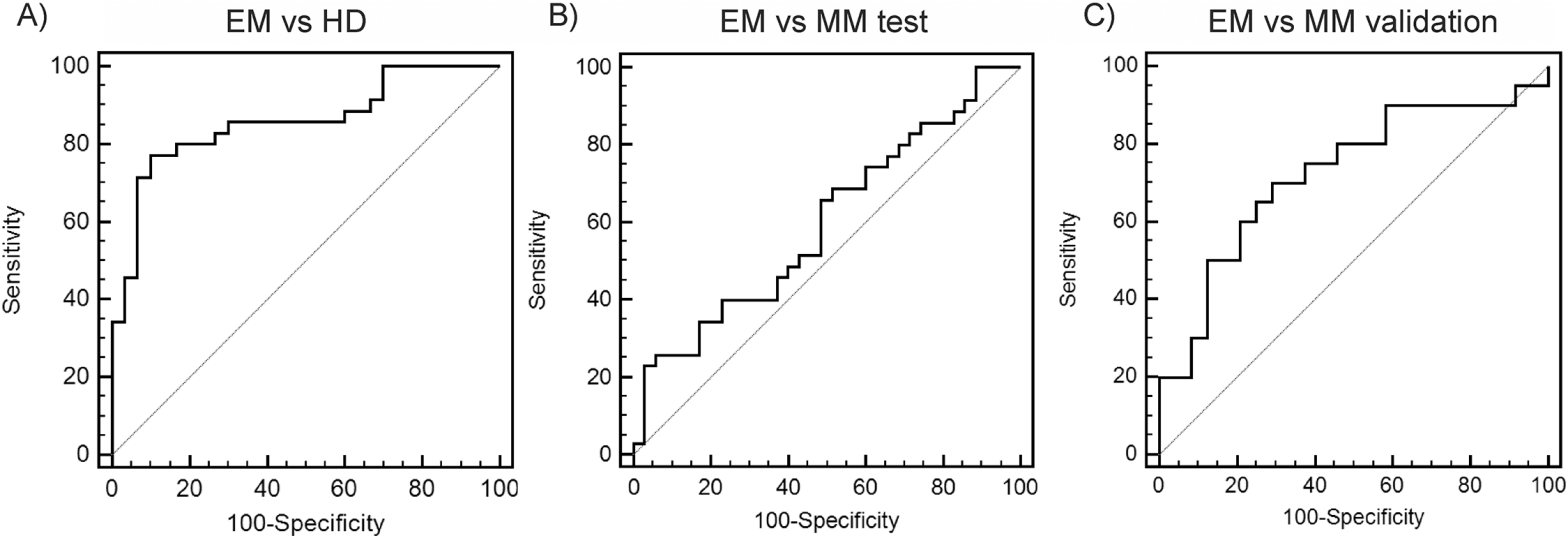
Receiver operating characteristic (ROC) analysis. ROC curves for miR-130a distinguishing **A)** extramedullary myeloma (EM) patients from healthy donors (HD) with area under the curve (AUC) = 0.856, **B)** EM patients from newly diagnosed multiple myeloma patients (MM new diagnosis) with AUC = 0.598 in a test cohort **C)** EM patients from MM patients (with PET/CT negative for EM) with AUC = 0.761 in a validation cohort.

To characterize miR-130a as a possible diagnostic marker for EM, receiver operating characteristic (ROC) analysis was performed (S2 Table). It revealed that miR-130a is potent to distinguish EM pts from HD with area under the curve (AUC) = 0.856, specificity of 90.0% (95%CI: 73.5–97.9) and sensitivity of 77.1% (95%CI: 59.9–89.6) using cut-off value = 0.0699. Most importantly, miR-130a is able to distinguish EM from newly diagnosed MM with AUC = 0.598, specificity of 77.1% (95%CI: 59.9–89.6) and sensitivity of 34.3% (95%CI: 19.1–52.2) using cut-off value = 0.041 in a test cohort. In the validation cohort miR-130a distinguished EM from MM pts without EM (newly diagnosed and relapsed) with AUC = 0.761 and using the same cut-off value from test cohort with specificity of 91.7% (95%CI: 73.0–99.0) and sensitivity of 31.6% (95%CI: 12.6–56.6) (Fig 2A, 2B and 2C).

### MiR-130a is associated with PCs infiltration, but does not have a prognostic value

Spearman bivariate correlation was performed to estimate association between clinical parameters of EM pts and miRNA levels. In EM test cohort (35 pts), serum miR-130a correlated negatively with percentage of PCs infiltration in the BM and positively with albumin and thrombocytes count (*p*<0.05; r_s_ = -0.416, r_s_ = 0.380 and r_s_ = 0.426, respectively). Such correlations were not found in validation cohort of EM patients, although there was a trend for statistical significance for negative correlation with PCs infiltration (Table 4). Indeed, overall comparison of EM pts (55 pts) showed no significant correlations with biochemical parameters but still a trend for negative association with percentage of PCs infiltration in the BM (*p* = 0.064; r_s_ = -0.285). Further, miR-130a levels were not associated with ISS, DS stage, DS substage, type of M-Ig or type of light chain or any of studied chromosomal aberrations in EM cohort of pts evaluated separately and in total. MiR-130a was verified as a possible indicator of OS in EM group of pts. However, univariate Cox proportional hazards survival model with one explanatory variable showed no prognostic impact on OS for this miRNA (data not shown).

**Table 4:** Correlation of serum miR-130a levels with clinically important biochemical parameters. Data are presented as coefficients of correlation. Statistically significant correlations are marked in bold and italics.

miR-130a correlation	Test cohort		Validation cohort	
r_S_	MM	EM	MM	EM
Monoclonal Ig (g/l)	- 0.201	- 0.217	0.259	0.067
Hemoglobin (g/l)	0.270	0.201	- 0.268	0.267
Thrombocytes (count x10^9^)	0.245	***0*.*426***	0.131	- 0.120
Calcium (mmol/l)	- 0.025	- 0.006	0.033	0.183
Albumin (g/l)	0.170	***0*.*380***	- 0.096	0.236
Creatinine (μmol/l)	- 0.129	0.050	0.150	- 0.123
β2-microglobulin (mg/l)	- 0.216	- 0.185	0.368	- 0.315
Lactate dehydrogenase (μkat/l)	0.048	- 0.082	0.045	- 0.010
C-reactive protein (mg/l)	- 0.176	- 0.322	0.007	- 0.069
Plasma cells infiltration of bone marrow (%)	0.046	***- 0*.*416***	- 0.075	- 0.325

### MiR-130a in BMPCs and tumor PCs

**Fig 3:**
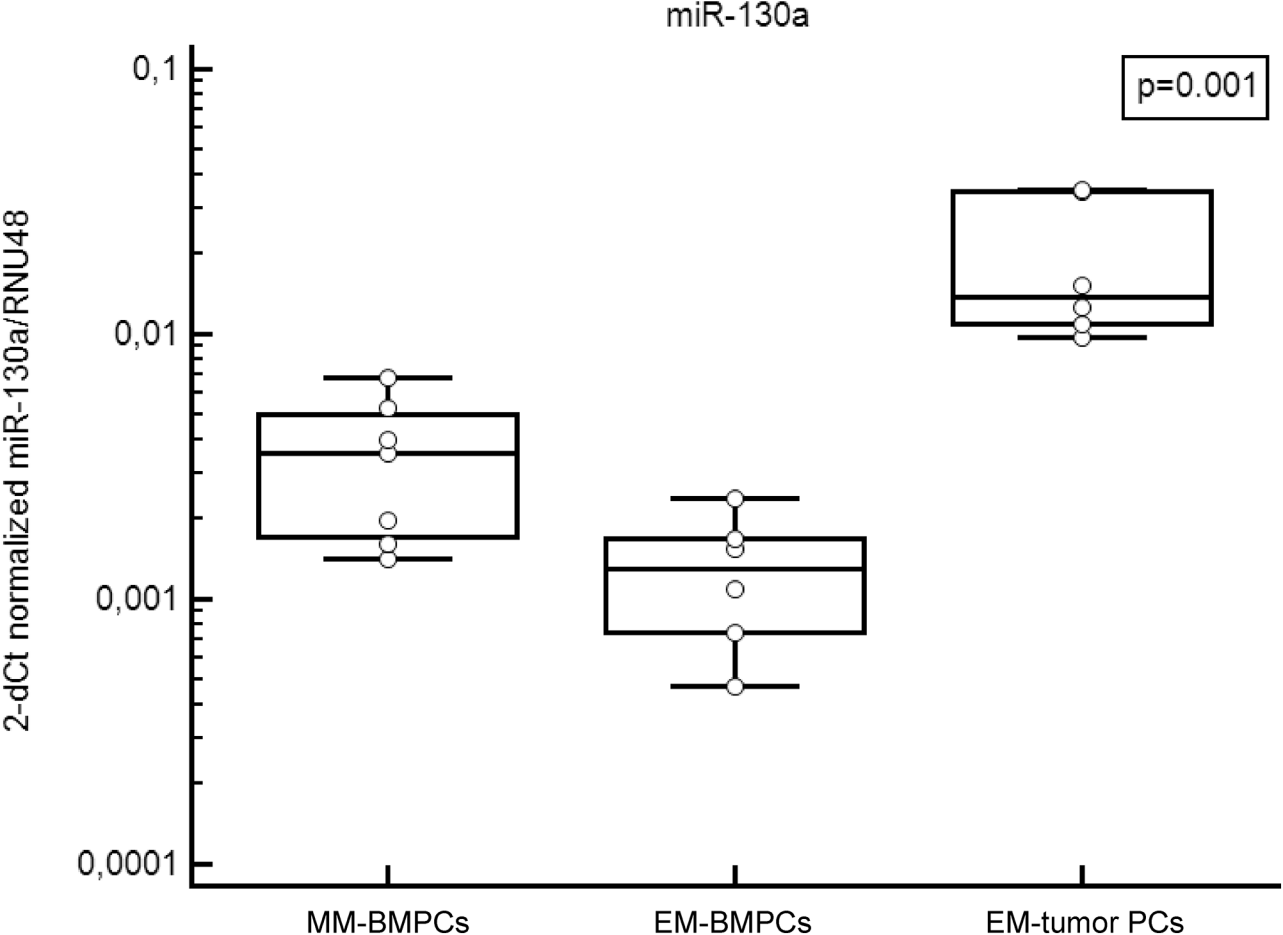
Level of miR-130a in CD138+ bone marrow plasma cells and tumor plasma cells. Level of miR-130a normalized to RNU48 in bone marrow plasma cells (BMPCs) and tumor plasma cells of multiple myeloma (MM) patients and patients with extramedullary disease (EM).

Since there was an association between serum miR-130a level and percentage of BMPCs infiltration in EM samples, level of miR-130a was evaluated in 7 relapsed samples of BMPCs of pts without EM (MM-BMPCs), 6 BMPCs of secondary EM pts (EM-BMPCs) and 6 samples of PCs obtained from EM tumor. Level of miR-130a was significantly decreased in EM-BMPCs as compared with MM-BMPCs (*p* = 0.035). However, there was not significant correlation between levels of miR-130a in BMPCs and serum (*p* = 0.208, r_s_ = 0.600), which, however, might be due to limited amount of samples. On contrary, level of miR-130a was significantly increased in PCs obtained from EM tumor in comparison with BMPCs of MM and EM pts (*p* = 0.001) (Fig 3).

## Discussion

Circulating miRNA hold great promise as a new class of diagnostic markers although their study remains challenging, due to inconsistent platforms used for their study as well as inconsistency in revealed deregulated miRNA and further evaluation [25]. Furtheremore, their origin, transit and location remains unknown. Previous reports of circulating miRNAs as diagnostic markers identified 3 miRNA: miR-720, miR-1308 and miR-1246 with diagnostic potential in MM [19]. Our previous report also revealed combination of miR-34a and let-7e to have a diagnostic potential for MM and monoclonal gammopathy of undetermined significance (MGUS) patients [20]. MiRNA hold a prognostic value as well, as was shown by us (miR-744 and let-7e) and others [20,26]. Others furtheremore reported miRNA, such as miR-16 and miR-25 to be independent prognosticators of OS [26] or a marker of high-risk MM (miR-19a) [27]. Signature of serum miRNA was also described to be a marker of progression-free survival after autologous stem-cell transplantation [28]. In this report, circulating miRNA with a potential to discriminate EM disease were studied. MiR-130a was evaluated by TLDA and further confirmed by qPCR in two independent cohorts of EM pts and MM pts as well as HD as being down-regulated in EM serum samples. This observation builds on our previous study, where we showed that lower levels of miR-130a were able to discriminate MM and MGUS serum samples from HD [20]. As serum miR-130a was also able to discriminate EM from newly diagnosed and relapsed/progressed MM pts without EM verified by PET/CT with high specificity, it makes it a new putative minimally-invasive marker of EM. We are aware that a prospective analysis of miR-130a on larger cohorts of MM pts who are negative by PET/CT as well as whole body MRI analyses for EM and on EM pts is needed; therefore, we will continue with further prospective validation. However; as nowadays PET/CT and whole body MRI are required for EM diagnosis [9,29]; estimation of miR-130a level in MM patients could point to a possible EM and thus streamline EM detection in such suspected pts.

In the group of EM pts, level of serum miR-130a was not significantly associated with biochemical parameters; however, lower miR-130a correlated with higher percentage of BMPCs infiltration. Such correlation indicates the connection between low serum miR-130a and the disease; however, the underlying biology remains to be explored. Our previous work showed that miR-130a is present in BMPCs of MM pts and in serum exosomes [20]. Present study further reveals that level of miR-130a is not only lower in serum, but also in BMPCs of EM pts; however, it is higher in PCs from extramedullary tumor in comparison with BMPCs (both MM-BMPCs and EM-BMPCs). The precise mechanism of action of miR-130a in MM is not known; however, there is growing evidence about the role of miR-130a in hematopoiesis and hematological malignancies. MiR-130a was recently shown to regulate C/EBP-ε expression during granulopoiesis, which is required for timed expression of secondary granule proteins and cell cycle exit. Specifically, cells with high miR-130a showed reduced proliferation, changed cell cycle regulation and altered maturation [30]. In chronic lymphocytic leukemia (CLL), miR-130a is among the most downregulated miRNAs and its enforced expression impaired cell viability and inhibited pro-survival autophagic activity via regulation of *ATG2B* and *DICER1* [31]. Indeed, decreased expression of DICER1, a regulator of miRNA biogenesis, led to downregulation of several miRNAs within the cell, possibly explaining the complexity of changes caused by miR-130a and also suggesting the presence of feedback loops and the involvement of other miRNAs. Given that in MM, as well as in CLL, accumulation of quiescent cells is primarily due to defects in apoptosis induction, rather than increased cell proliferation, at least partial involvement of miR-130a in MM pathogenesis is possible.

Since miRNA can act as signaling molecules [12], it can be speculated that miR-130a is actively uptaken from circulation by extramedullary tumor site to support angiogenesis in the microenvironment of ‘escaped’ PCs. Although we cannot draw any conclusion from that, we noted higher levels of miR-130a in endothelial cell lines compared to MM PCs and MM cell lines, suggesting role of miR-130a in endothelial cells and thus we continue further research in this field. Indeed, miR-130a has been previously described in endothelial cells to have pro-angiogenic properties as it is an important regulator of *GAX* and to a lesser extent *HOXA*, homeobox genes involved in inhibition of angiogenesis in endothelial cells [32]. Thus, downregulation of miR-130a in PCs and its upregulation in surrounding endothelial cells might be involved in MM pathogenesis. However, despite all insights in this field, only little is known about the origin of circulating miR-130a.

In summary, this is the first report describing circulating miRNA in patients with EM. Specifically, miR-130a was decreased in serum samples of pts developing EM disease as compared with newly diagnosed, relapsed and progressed MM pts in two independent cohorts of pts. Moreover, miR-130a was able to distinguish EM pts from MM pts with specificity over 77%. Thus, our results suggest that levels of miR-130a below cut-off 0.041 (normalized to miR-19b) might provide a new minimally-invasive biomarker of diagnostic value for EM disease in MM which; however, need to be further validated.

## Supporting Information

S1 Fig Repeatability of miRNA/RNA extraction and qPCR assay. (DOCX)

S1 Table Baseline characteristics of patients and healthy donors used in TaqMan Low Density Arrays analysis. (DOCX)

S2 Table Receiver operating characteristic (ROC) analysis of miR-130a. (DOCX)
