# Supplementary material for: Circulating Serum MicroRNA-130a as a Novel Putative Marker of Extramedullary Myeloma
Source: PLoS One. 2015 Sep 21;10(9):e0137294. doi: 10.1371/journal.pone.0137294 (PMC4577078; doi:10.1371/journal.pone.0137294)

**S1 Figure. Repeatability of miRNA/RNA extraction and qPCR assay.** **A)** The repeatability of miRNA/RNA extraction was assessed using pooled serum from 20 healthy donors that was divided into two equal portions (each 400µl). Total miRNA/RNA was extracted from these two pooled serum samples, respectively. Further, amounts of fifteen miRNAs (miR-29a, miR-21, miR-130a, miR-19b, miR-16, miR-34a, let-7a, miR-222, miR-320B, miR-320, miR-151-5P, miR-483-5P, miR-221, miR-106a and miR-195) were determined in triplicates using TaqMan stem-loop primers and TaqMan primer-probe qPCR assays. MiRNAs levels, presented as Cq values from triplicates were plotted against each other. Coefficient of correlation between the two replicates is included. **B)** The repeatability of qPCR assay was assessed using detection of the same fifteen miRNAs as above in the two same miRNA/RNA samples extracted from pooled serum from 20 healthy donors. Similarly, the Cq values of the miRNAs from triplicates were plotted against each other and coefficient of correlation between the two replicates is included as well.


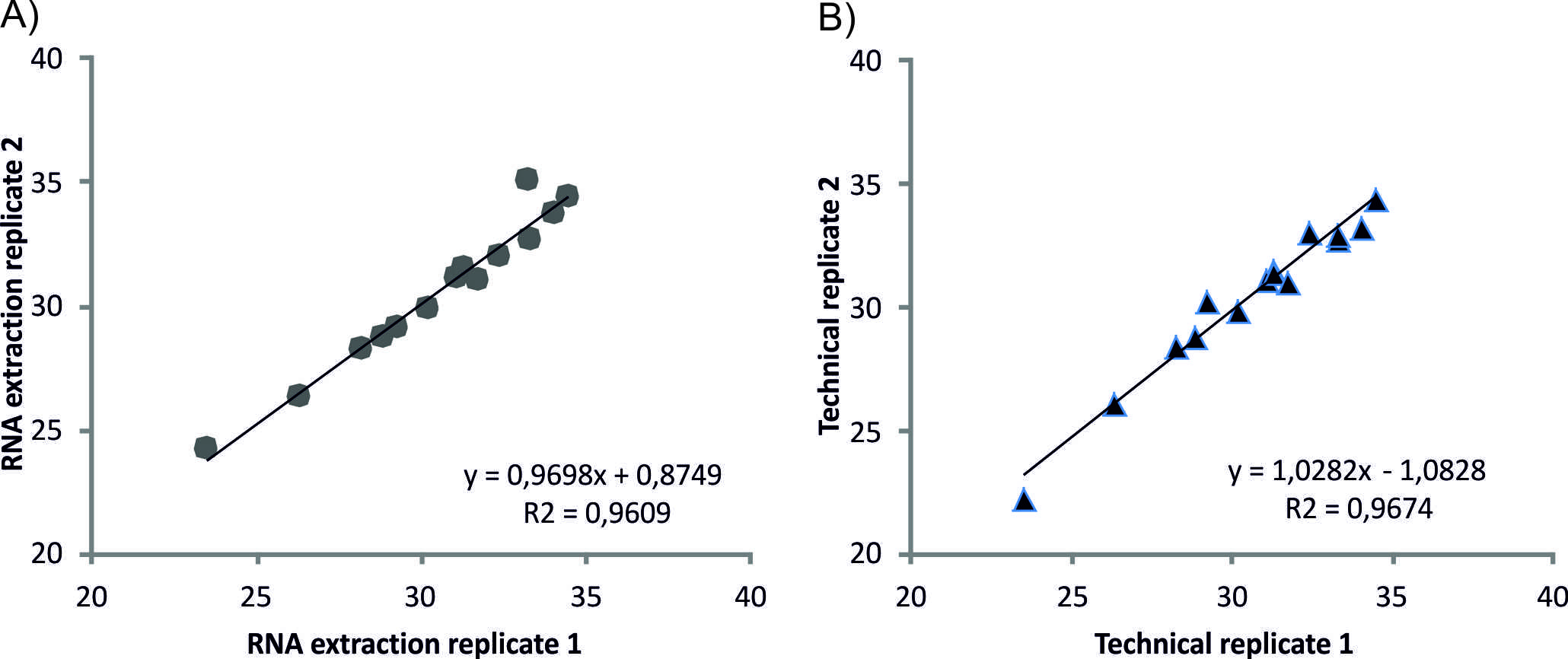

Supplement: S1 Fig — (DOCX) [file pone.0137294.s001.docx]
