# Supplementary material for: Circulating Serum MicroRNA-130a as a Novel Putative Marker of Extramedullary Myeloma
Source: PLoS One. 2015 Sep 21;10(9):e0137294. doi: 10.1371/journal.pone.0137294 (PMC4577078; doi:10.1371/journal.pone.0137294)
Supplement: S1 Table — (DOCX) [file pone.0137294.s002.docx]

**S1 Table. Baseline characteristics of patients and healthy donors used in TaqMan Low Density Arrays analysis.** Clinical characteristics of healthy donors (HD), multiple myeloma patients (MM) and patients with extramedullary myeloma (EM) used in TLDA analysis.

|  | HD | MM | EM |
| --- | --- | --- | --- |
| **No. of patients/donors** | **6** | **5** | **5** |
| Gender: males-females | 50%-50% | 60%-40% | 60%-40% |
| Age median (min-max) [years] | 56 (54-64) | 70 (53-83) | 57 (54-68) |
| ISS stage: I-II-III | ND | 20%-40%-40% | 67%-0%-33% |
| Durie-Salmon stage: I-II-III | ND | 0%-20%-80% | 0%-0%-100% |
| Durie-Salmon substage: A-B | ND | 80%-20% | 100%-0% |
| Ig isotype: IgG-IgA-NonSecretory | ND | 60%-20%-20% | 60%-20%-20% |
| Light chains: kappa-lambda | ND | 25%-75% | 50%- 50% |
| **Type of EM** |  |  |  |
| Bone related | ND | 0% | 48% |
| Bone unrelated | ND | 0% | 36% |
| Both types | ND | 0% | 16% |
| **No. of previous treatment lines** |  |  |  |
| None | ND | 100% | 0% |
| 1-2-3-4 | ND | 0% | 20%-20%-60% |
| **Biochemical parameters:** |  |  |  |
| **median (min-max)** |  |  |  |
| *Hemoglobin (g/l)* | *ND* | *108.0 (89.7-125)* | *110.0 (75.0-130.0)* |
| *Thrombocytes (count x10^9^)* | *ND* | *175.0 (117.0-334.0)* | *151.0 (59.0-160.0)* |
| *Calcium (mmol/l)* | *ND* | *2.29 (2.23-3.59)* | *2.36 (2.30-2.36)* |
| *Albumin (g/l)* | *ND* | *33.3 (28.0-41.7)* | *37.4 (36.9-44.7)* |
| *Creatinine (umol/l)* | *ND* | *99.0 (72.0-439.0)* | *95.0 (57.0-169.0)* |
| *β2-microglobulin (mg/l)* | *ND* | *5.41(3.02-6.66)* | *3.23 (2.77-5.52)* |
| *Lactate dehydrogenase (ukat/l)* | *ND* | *3.50 (2.20-4.13)* | *5.64 (4.07-17.95)* |
| *C-reactive protein (mg/l)* | *ND* | *7.50 (4.30-23.80)* | *7.20 (3.30-33.40)* |
| *Monoclonal Ig (g/l)* | *ND* | *25.0 (0-66.3)* | *18.6 (0-51.1)* |
| *Plasma cell infiltration of bone marrow (%)* | *ND* | *18.9 (14.80-26.4)* | *5.6 (0.4-96.6)* |
| **Chromosomal abnormality in BMPCs** |  |  |  |
| *13q14 deletion* | *ND* | *2 (40%)* | *3 (60%)* |
| *17p13 deletion* | *ND* | *0 (0%)* | *1 (20%)* |
| *1q21 gain* | *ND* | *1 (20%)* | *1 (20%)* |
| *IgH disruption* | *ND* | *2 (40%)* | *3 (60%)* |
| *Translocation t(4;14)* | *ND* | *1 (20%)* | *1 (20%)* |
| *1p36 deletion* | *ND* | *2 (40%)* | *2 (40%)* |
| *Hyperdiploidy* | *ND* | *1 (20%)* | *2 (40%)* |

ND = not determined
