# Supplementary material for: Circulating Serum MicroRNA-130a as a Novel Putative Marker of Extramedullary Myeloma
Source: PLoS One. 2015 Sep 21;10(9):e0137294. doi: 10.1371/journal.pone.0137294 (PMC4577078; doi:10.1371/journal.pone.0137294)
Supplement: S2 Table — (DOCX) [file pone.0137294.s003.docx]

**S2 Table. Receiver operating characteristic (ROC) analysis of miR-130a.** Sensitivity, specificity, 95% intervals for specificity and sensitivity and area under the curve (AUC) is presented for miR-130a in studied cohorts using defined cut-off value. Also positive (+) and negative (-) likehood ratios (LR) are presented. EM = patients with extramedullary disease, MM = multiple myeloma, HD = healthy donors.

| miR-130a | Sensitivity | 95%CI | Specificity | 95%CI | AUC | cut-off | +LR | -LR |
| --- | --- | --- | --- | --- | --- | --- | --- | --- |
| **EM/HD** | 77.1 | 59.9-89.6 | 90.0 | 73.5-97.9 | 0.856 | 0.0699 | 7.71 | 0.25 |
| **EM/MM Test**  (EM/MM new diagnosis) | 34.3 | 19.1-52.2 | 77.1 | 59.9-89.6 | 0.598 | 0.041 | 1.50 | 0.85 |
| **EM/MM Validation**  (EM/MM negative for EM) | 31.6 | 12.6-56.6 | 91.7 | 73.0-99.0 | 0.761 | 0.041 | 3.79 | 0.75 |
